# HuR and TIA1/TIAL1 Are Involved in Regulation of Alternative Splicing of SIRT1 Pre-mRNA

**DOI:** 10.3390/ijms15022946

**Published:** 2014-02-20

**Authors:** Wenhui Zhao, Jinfeng Zhao, Miaomiao Hou, Yue Wang, Yang Zhang, Xin Zhao, Ce Zhang, Dawei Guo

**Affiliations:** 1 Department of Neurobiology, Shanxi Medical University, 56# Xin Jian South Road, Taiyuan 030001, Shanxi, China; E-Mails: wenhui8088@gmail.com (W.Z.); jinfeng801229@gmail.com (J.Z.); houmiaomiaodeshijie@gmail.com (M.H.); fairymoon0325@gmail.com (Y.W.); marilyn2485208958@gmail.com (Y.Z.); xinzhao2006@gmail.com (X.Z.); 2 School of Forensic Medicine, Shanxi Medical University, 56# Xin Jian South Road, Taiyuan 030001, Shanxi, China

**Keywords:** SIRT1, alternative splicing, SIRT1-ΔExon8, Hu antigen R, T-cell-restricted intracellular antigen 1, TIA1-like 1

## Abstract

SIRT1 is a pleiotropic protein that plays critical and multifunctional roles in metabolism, senescence, longevity, stress-responses, and cancer, and has become an important therapeutic target across a range of diseases. Recent research demonstrated that SIRT1 pre-mRNA undergoes alternative splicing to produce different isoforms, such as SIRT1 full-length and SIRT1-ΔExon8 variants. Previous studies revealed these SIRT1 mRNA splice variants convey different characteristics and functions to the protein, which may in turn explain the multifunctional roles of SIRT1. However, the mechanisms underlying the regulation of SIRT1 alternative splicing remain to be elucidated. Our objective is to search for new pathways that regulate of SIRT1 alternative splicing. Here we describe experiments showing that HuR and TIA1/TIAL1, two kinds of RNA-binding proteins, were involved in the regulation of alternative splicing of SIRT1 pre-mRNA under normal and stress circumstances: HuR increased SIRT1-ΔExon8 by promoting SIRT1 exon 8 exclusion, whereas TIA1/TIAL1 inhibition of the exon 8 exclusion led to a decrease in SIRT1-ΔExon8 mRNA levels. This study provides novel insight into how the alternative splicing of SIRT1 pre-mRNA is regulated, which has fundamental implications for understanding the critical and multifunctional roles of SIRT1.

## 1. Introduction

SIRT1, known as III histone deacetylases (HDACs), has been shown to participate in a variety of physiopathological processes including health maintenance in development, gametogenesis, homeostasis, longevity, and several neurodegenerative diseases as well as age-related disorders [1–5]. Activation of SIRT1 generally displays beneficial effects on regulation of oxidative stress, inflammation, cellular senescence, metabolism, autophagy, apoptosis, differentiation, and autoimmunity. Recently, SIRT1 pre-mRNA was reported to be subject to alternative splicing, and the different SIRT1 splice variants have been shown to have distinct characteristics and functions [6,7]. The SIRT1-ΔExon8 isoform was the first reported SIRT1 splice variant, in which the 558 bp region of exon 8 was precisely excluded. Exon8 encodes the deacetylase domain, which is critical for SIRT1 function. The exclusion of exon 8 results in altered functions including reduced neuroprotective effect as compared to the full-length SIRT1 (SIRT1-FL) [6]. The mechanisms underlying the regulation of alternative splicing of SIRT1 pre-mRNA, however, remain unclear.

It is well known that in mammalian cells, pre-mRNA splicing, as well as other aspects of mRNA processing are largely governed by RNA-binding proteins (RBPs) which interact with pre-mRNAs forming pre-mRNA-protein complexes so as to regulate splicing, capping, 3′ end formation, surveillance, nucleocytoplasmic transport, storage, stability, decay and translation [8–10]. Therefore, RBPs play key roles in post-transcriptional gene expression regulation and further regulate cellular processes under normal circumstances, and stress, or in disease [11–14]. Hu antigen R (HuR), a ubiquitously expressed member of the Hu/ELAV-like family is probably the most extensively investigated RBP [15]. Besides HuR there are other three neuro-specific members of HuB, HuC, and HuD in this family [16–18]. HuR is predominantly located in the nucleus, although it can translocate to the cytoplasm where it interacts with the target mRNAs (through U/AU-rich elements), modulating post-transcriptional processes, and further influencing the cellular functional responses [19–23]. Recently it was reported that HuR was involved in regulation of alternative splicing, in which HuR usually promoted variable exon skipping by inhibiting the association of U2 snRNP auxiliary factor 65 kDa (U2AF65) with the upstream 3′ splice site or by preventing the binding of U1 and U6 snRNPs to the downstream 5′ splice site, or alternatively by accelerating the local transcriptional elongation rate upon binding to its target sequence [24–29].

The T-cell-restricted intracellular antigen (TIA) family is another important kind of RBPs and has two members, TIA1 and TIA1-like 1 (TIAL1; also referred to as TIAR). Importantly, they share very similar domain architectures with HuR, and all of them have three RNA recognition motifs (RRMs) [17,30–33]. TIA1/TIAL1 usually destabilize their target mRNAs or inhibit their translation of their target mRNAs [34,35]. TIA1/TIAL1 are also reported to act as alternative splicing regulators, in which they promote variable exon inclusion by advancing the combination of U1 and U6 snRNPs to the 5′ splice site upon binding to U-rich sequences in the pre-mRNA [24,26–28,36]. Therefore HuR and TIA1/TIAL1 can usually act sequentially on shared target mRNAs by competing to one same combining site (U/AU-rich motifs), cooperating to regulate mRNA metabolism including the process of pre-mRNA alternative splicing [24,26–28].

Accordingly, alternative splicing of SIRT1 pre-mRNA produced different isoforms leading to substantial changes of biological functions; exploring the mechanisms underlying the alternative splicing of SIRT1 is thus particularly imperative. Based on the analysis of SIRT1 gene sequence, we found that some AT/T rich domains exist around the exon 8 region; it was reasonable to presume that HuR or TIA1/TIAL1 might bind to these domains and regulate the alternative splicing of SIRT1 pre-mRNA. The present study was therefore designed to investigate whether HuR or/and TIA1/TIAL1 were involved in regulating SIRT1 pre-mRNA alternative splicing so as to affect SIRT1-ΔExon8 mRNA levels under normal as well as under injurious circumstances, such as exposure to UV radiation.

## 2. Results and Discussion

### 2.1. HuR Promotes Exclusion of SIRT1 Exon 8

**Figure 1.**
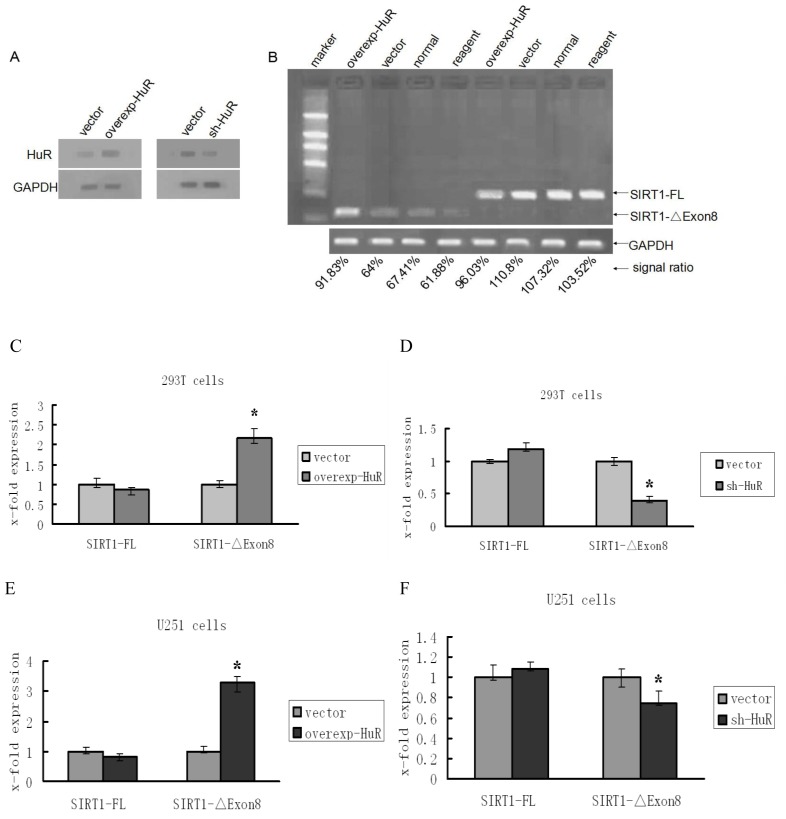
HuR regulates the SIRT1-ΔExon8 mRNA levels in 293T cells and U251 cells. (**A**) The changes of HuR protein levels after HuR overexpression or silencing in 293T cells; (**B**) PCR products were separated in 1% agarose gels, the arrows point at the location of SIRT1-FL and SIRT1-ΔExon8 corresponding to the 200 and 111 bp of the left mark, respectively; GAPDH was used as the loading control, the signal ratio was calculated as the ratio of signal for the SIRT-FL or SIRT1-ΔExon8 band and the GAPDH band; (**C**–**F**) Splice-variant-specific quantitative Real-Time PCR (qRT-PCR) of SIRT1-FL and SIRT1-ΔExon8 were shown in 293T cells and U251 cells. The levels of SIRT1-ΔExon8 mRNA were increased after HuR over-expression to 2.17 fold in 293T cells (**C**) and 3.29 fold in U251 cells (**E**). The interference of HuR resulted in the notable reduction of SIRT1-ΔExon8 with 62% in 293T cells (**D**) and 28% in U251 cells (**F**); ^*^ *p* < 0.05 compared to corresponding control.

In order to ascertain whether HuR is involved in the regulation of SIRT1-ΔExon8 mRNA level, we transfected a HuR over-expression plasmid into 293T cells, and measured the levels of SIRT1-FL and SIRT1-ΔExon8 by PCR and real-time PCR. With the high transfection efficiency, the levels of HuR protein were significantly increased in HuR over-expression cells and obviously decreased in HuR depletion cells (Figure 1A). A selective increase of SIRT1-ΔExon8 mRNA was observed when over-expressed HuR in 293T cells (Figure 1B,C). In knockdown experiments, the results showed that depletion of HuR significantly decreased the level of the SIRT1-ΔExon8 isoform (Figure 1D). The same experiments were also carried out in U251 cells and similar results were obtained (Figure 1E,F).

**Figure 2.**
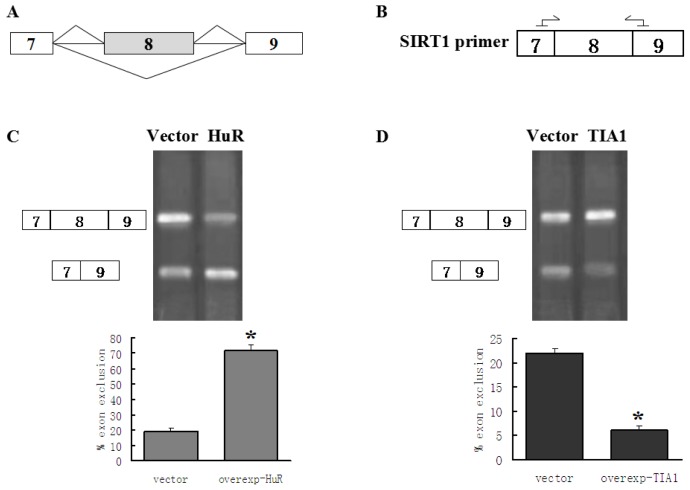
HuR and TIA1/TIAL1 regulate alternative splicing of SIRT1 exon 8 of SIRT1 minigene; (**A**) Schematic structure of SIRT1 minigene, shaded boxes are alternatively spliceable exons; (**B**) The arrows point at the location of the primers of SIRT1; (**C**) HuR caused a significant increase in exon-exclusion for SIRT1 RNAs; (**D**) TIA1 induced SIRT1 minigene exon 8 inclusion. The splicing band patterns were quantified using Image J software [38] and the results showed as below. The results obtained from three independent experiments. ^*^ *p* < 0.05 compared to co-transfect empty plasmid and SIRT1 minigene.

Previous studies reported that HuR regulates mRNA expression and thus affects the level of mRNA by enhancing the stability of mRNA [37]. To confirm whether the regulatory effects of HuR on the SIRT1-ΔExon8 level was due to regulation of pre-mRNA alternative splicing other than promotion of SIRT1-ΔExon8 stability, we constructed a SIRT1 minigene which only contained alternatively spliceable exon 8 flanked by exons 7 and 9 with their respective introns (Figure 2A). 293T cells were co-transfected with the minigene and a HuR construct or an empty vector. By using the primers (sense sequence in exon 7 of SIRT1, and antisense sequence in exon 9 of SIRT1, Figure 2B) that specifically amplified either the fragment containing exon 8 sequences or the one that does not contain them, two variants of the SIRT1 minigene were obtained by PCR examination. Agarose gel band densitometry results showed a significant increase in exon 8 exclusion when HuR over-expressed (Figure 2C). Consequently, both the endogenous and exogenous SIRT1-ΔExon8 mRNA detections provided solid evidence of HuR for modulation of the alternative splicing of SIRT1 pre-mRNA by promotion of exon 8 exclusion.

There are two points worth noting when interpreting the observed regulatory effects of HuR on alternative splicing of SIRT1 exon 8 in both 293T and U251 cell lines. The first point is that although the quantity of HuR cDNA plasmid transfected into U251 cells was small compared with that transfected into 293T cells (data not shown), the influence on the SIRT1 alternative pattern was greater in U251 cells than in 293T cells (Figure 1C,E). The possible reasons might be attributed to the diversity expression and distribution patterns of Hu family members in different cells. For four members of Hu family, only HuR was expressed in 293T cells, whereas all four Hu family members (HuR, HuB, HuC, and HuD) were expressed in U251 cells [15–18]. Moreover, HuR and the other three Hu family members can interact with each other resulting in an alteration of their individual efficiencies [29,39,40]. Potential regulatory roles of HuR in U251 cells may be augmented, in this case, by other Hu proteins. Similarly, in the HuR depletion observation, when HuR was down regulated, the other three Hu proteins might contribute to some extent in the regulation, so that, although the depletion of HuR was apparent, the decrease of SIRT1-ΔExon8 was not as sharp as compared with the change in 293T cells (Figure 1D,F). The second point is that HuR promoted SIRT1-ΔExon8 mRNA level. However, it had little effect on SIRT1-FL mRNA (Figure 1B–F). Generally, if HuR regulates SIRT1-ΔExon8 mRNA level via alternative splicing mechanism, a reduction in the SIRT1-FL mRNA should be observed whenever the SIRT1-ΔExon8 mRNA level increased. There might be two reasons to explain these observations: (i) In naïve 293T and U251 cells, the isoforms of SIRT1-FL are predominant, and the levels of SIRT1-ΔExon8 mRNA are very low. It is reasonable to assume when SIRT1 pre-mRNA undergoes alternative splicing induced by HuR, the SIRT1-ΔExon8 level would be greatly increased, whereas the level of SIRT1-FL isoform may just show undetectable change in this case; (ii) HuR, on one hand promotes SIRT1 exon 8 skipping to decrease SIRT1-FL, and on the other hand increases the stability of SIRT1-FL [37]; these opposite effects may neutralize each other leading to no apparent variation of SIRT1-FL mRNA isoforms.

### 2.2. TIA1/TIAL1 Inhibits Exclusion of SIRT1 Exon 8

**Figure 3.**
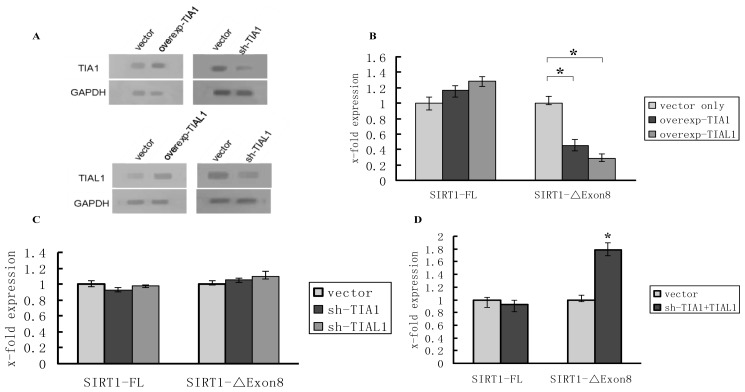
TIA1/TIAL1 regulates the SIRT1-ΔExon8 mRNA levels in 293T cells detected by Real-Time PCR. (**A**) The TIA1/TIAL1 protein levels after TIA1/TIAL1 overexpression or silencing in 293T cells; (**B**) Over-expression of TIA1/TIAL1 decreased the levels of SIRT1-ΔExon8 for about 60% and 70%, respectively; (**C**) The levels of SIRT1-FL and SIRT1-ΔExon8 mRNA had no significant differences after depletion of either TIA1 or TIAL1; (**D**) Reducing the levels of TIA1 and TIAL1 simultaneously resulted in SIRT1-ΔExon8 increase. ^*^ *p* < 0.05 compared to transfect corresponding vector control.

As mentioned previously, HuR and TIA1/TIAL1 usually compete to one same combining site (U/AU-rich motifs) and function jointly on shared target mRNAs to regulate their metabolism [24,26–28], we hypothesized that TIA-1/TIAL1 may also function as a SIRT1 pre-mRNA alternative splicing regulator by inhibiting exon 8 exclusion. To test this hypothesis, over-expression and shRNA knockdown experiments were carried out in 293T cells. The levels of TIA1/TIAL1 protein were significantly increased in TIA1/TIAL1 over-expression cells and obviously decreased in TIA1/TIAL1 depletion cells (Figure 3A). Not surprisingly, over-expression of TIA1/TIAL1 led to a decrease in the levels of SIRT1-ΔExon8 mRNA (Figure 3B). However, when depleted of TIA-1 or TIAL1 individually by shRNA, the level of SIRT1-ΔExon8 was not significant changed (Figure 3C), whereas the mRNA level of SIRT1-ΔExon8 significantly increased when both of them were reduced simultaneously (Figure 3D). These results indicate that TIA1 and TIAL1 might share redundant function to regulate SIRT1-ΔExon8 mRNA levels, in other words, even if only one of either TIA1 or TIAL1 functions it is sufficient to promote inclusion of SIRT1 exon 8, thereby preventing increase of SIRT1-ΔExon8 mRNA.

In addition, for the same reason stated above, we carried out minigene transfection experiment to confirm the regulatory effect of TIA1 on SIRT1-ΔExon8, in which transiently transfected SIRT1 minigene plasmids along with over-expressed TIA1 plasmids or empty vector into 293T cells, and tested the status of exogenous SIRT1-ΔExon8 and SIRT1-FL mRNAs. The results showed that over-expression of TIA1 led to a decrease in SIRT1-ΔExon8 mRNA (Figure 2D).

Taking the above results together, we conclude that both HuR and TIA1/TIAL1 are involved in regulation of SIRT1 pre-mRNA alternative splicing. HuR regulates the production of the short isoforms of SIRT1 by promotion of SIRT1 exon 8 exclusion, whereas TIA-1/TIAL1 inhibit exclusion of SIRT1 exon 8 leading to a decrease in SIRT1-ΔExon8 mRNA levels. However, due to the diverse mechanisms to modulate SIRT1 mRNA isoform, we cannot rule out that there may be other mechanisms possibly involved, such as the stability of the mRNAs, and the degree and rates of the mRNAs’ degradation, which additional studies may clarify.

### 2.3. HuR and TIA1/TIAL1 Regulate the Alternative Splicing of SIRT1 Exon 8 under Injury Circumstance

**Figure 4.**
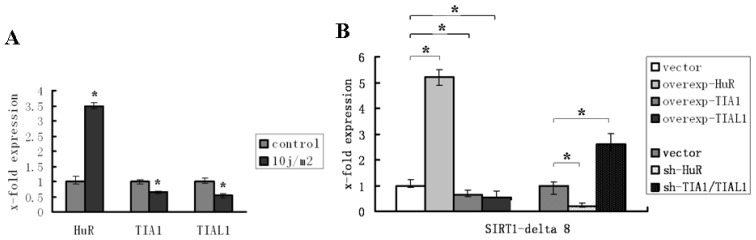

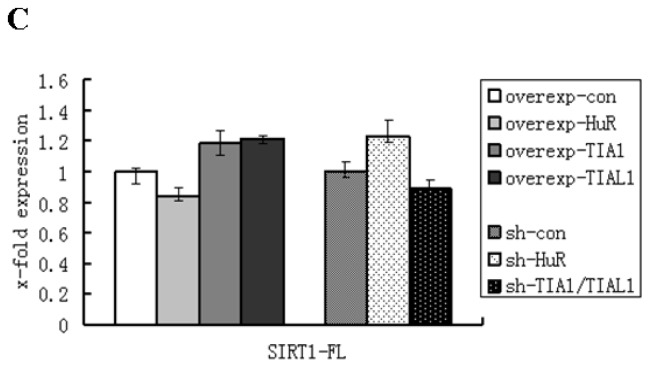
HuR and TIA1/TIAL1 regulated the alternative splicing of SIRT1 exon 8 in UV injury model. (**A**) The changes of HuR and TIA1/TIAL1 mRNAs under UV injury (10 j/m^2^) condition. The level of HuR was markedly increased after UV exposure, while the level of TIA1 or TIAL1 was decreased; (**B**) The change of SIRT1-ΔExon8 mRNA after HuR or TIA1/TIAL1 over-expression or depletion respectively under UV injury condition; (**C**) The levels of SIRT1-FL mRNA no significant change. ^*^ *p* < 0.05 compared to transfect corresponding vector control.

Previous experiments in our lab had demonstrated that ultraviolet exposure induced SIRT1 exon 8 exclusion and led to an increase of SIRT1-ΔExon8 mRNA level [41]. In order to explore whether HuR and TIA1/TIAL1 were involved in regulation of the change of SIRT1-ΔExon8 mRNA level under this stress condition, as they do under normal circumstance, a UV injury model was used. We observed that the level of HuR increased when cells were exposed to UV, whereas the levels of TIA1/TIAL1 decreased (Figure 4A). The underlying mechanism for this phenomenon may be at least partially attributable to the regulatory effects of HuR and TIA1/TIAL1 on SIRT1 pre-mRNA alternative spicing. In other words, injurious stimulation induced increase of HuR as well as decrease of TIA1/TIAL1; their integrative regulatory effects on SIRT1 alternative splicing resulted in elevation of SIRT1-ΔExon8.

The interference observations confirmed these results, in which over-expression or knockdown of HuR or TIA1/TIAL1 experiments were carried out before UV exposure, and SIRT1-ΔExon8 mRNA levels were assessed, respectively. The results showed that the mRNA levels of SIRT1-ΔExon8 were significantly increased after HuR over-expression or TIA1/TIAL1 knockdown and were decreased when accompanied with HuR knockdown or TIA1/TIAL1 over-expression (Figure 4B). The levels of SIRT1-FL mRNA showed no significant change with over-expression or knockdown of HuR or TIA/TIAL1 (Figure 4C). Accordingly, we suggest that HuR and TIA1/TIAL1 participate to modulate the alternative splicing of SIRT1 pre-mRNA under UV-induced stress conditions.

## 3. Experimental Section

### 3.1. Cell Culture and Generation of Cell Lines

293T and U251 cells were obtained from ATCC and were cultured in Dulbecco’s modified Eagle’s medium (DMEM) (Hycolone, Watham, MA, USA) supplemented with 10% fetal bovine serum (FBS, TransGen, Beijing, China) and 1% Penicillin/Streptomycin (BOSTER, Wuhan, Hubei, China).

### 3.2. shRNA and Plasmids

A generic scrambled sequence negative control shRNA cloned into the psi-H1 vector for RNA interference was purchased from GeneCopoeia (Guangzhou, Guangdong, China). The knockdown plasmids (also from GeneCopoeia, Guangzhou, Guangdong, China) have the following target sense sequences matching the corresponding genes: HuR/ELAVL1: 5′-aaggacgtagaagacatgt-3′, TIA1: 5′-aagctctaattctgcaactct-3′ and TIAL1: 5′-aaccatggaatcaacaaggat-3′.

Plasmids pReceiver-M29-ELAVL1/HuR, pEZ-M29-TIA1, pEZ-M29-TIAL1 constructs were made by inserting the corresponding cDNAs. Sequence analysis showed a full match to the sequenced published by the NCBI (ELAVL1/HuR: NM_001419.2, TIA1: NM_022173.2, TIAL1: NM_003252.3). The inserts from the plasmids above were subcloned into pEZ-M29. The empty vector pEZ-M29 was used as a negative control.

The SIRT1 minigene was made by inserting a fragment of the SIRT1 cDNA (NCBI: NM_012238.4) encompassing only the alternatively spliceable exon 8 flanked by exons 7 and 9 and the corresponding introns (GeneCopoeia, Guangzhou, Guangdong, China).

### 3.3. Transfection

All of the plasmids and shRNAs were transfected with Attractene Transfection Reagent (Qiagen, Shanghai, China). Transfections were performed according to the standard manufacturer’s protocol at a 3:1 Attractene to DNA ratio.

### 3.4. Western Blot Analysis

Cells for analysis were washed three times with PBS, and 1 mL 1× PBS was added to every well of the six-well plates. Cells were collected in 1.5 mL EP tubes, centrifuged at 2000 rpm for 5 min, the supernatant aspirated, 200 μL cell lysates (Beyotime, No.: P0013, Shanghai, China) added, mixing, 30 min on ice, centrifuged at 12,000 rpm for 10 min. Take 10 μL supernatant, add 2 μL 6× protein sample buffer, mix, 95 °C constant temperature bath for 5 min, place on the ice for electrophoresis. SDS-PAGE prepared according to the kit illustration (Beyotime, No.: P0012A, Shanghai, China). Protein concentrations determined using a bicinchoninic (BCA) protein assay kit (Thermo Scientific, Wilmington, DE, USA). Protein samples were resolved on the 10% SDS-PAGE gel and transferred on a nitrocellulose membrane. The following primary and secondary antibodies were used: rabbit polyclonal anti-HuR antibody (Proteintech Group, Inc., Catalog No.: 11910-1-AP, Chicago, IL, USA), rabbit polyclonal anti-TIA1 antibody (Proteintech Group, Inc., Catalog No.: 12133-2-AP, Chicago, IL, USA), rabbit polyclonal anti-TIAL1 antibody (Proteintech Group, Inc., rabbit, NO.: 17649-1-AP, Chicago, IL, USA), Horseradish Peroxidase-conjugated AffiniPure Goat Anti-Rabbit IgG (H + L) (Jackson NO.: 111-035-003, Lancaster, PA, USA).

### 3.5. RNA Analysis

Total RNA was isolated using Trizol reagent following the manufacturer’s recommendations. To generate cDNA, reverse transcription was carried out using a PrimeScript^®^ RT Master Mix (TaKaRa, Dalian, Liaoning, China). Generally, 0.8 to 3 μg of total RNA was used per 20 μL of RT reaction mixture, reverse conditions were 37 °C for 15 min, 85 °C for 5 min, 4 °C 1 h.

For the SIRT1 minigene, reverse transcription was carried out using the primer, 5′-atcaaacaaatcatagctcgagtgcgg-3′ (span the junction flank the vector and the exon 9 of SIRT1) and TransScript First-Strand cDNA Synthesis SuperMix (TransGen, Beijing, China), reverse conditions were 42 °C for 30 min, 85 °C for 5 min. PCR was performed using specific SIRT1 primers, 5′-CCG CTT GCT ATC ATG AAA CCA-3′ (forward, in SIRT1 exon 7), 5′-TCT CCA TCA GTC CCA AAT CCA-3′ (reverse, in SIRT1 exon 9), 94 °C for 3 min, 34 cycles of 94 °C for 30 s, 60 °C for 30 s, 72 °C for 4 min and followed by 72 °C for 8 min. PCR products were separated in 1% agarose gels and stained with Gold View I (Solarbio, Beijing, China). The splicing band pattern was quantified using Image J software [38]. Percentage exon exclusion was calculated as the ratio of signal for the lower band (the shorter one, only exons 7 and 9) and sum of signals for upper (the longer one, contain exons 7, 8 and 9) and lower bands. Statistical analysis was performed using SPSS and analysis of variance corrected by Dunnett’s test for repeated measures.

Semiquantitative PCR was carried out in the linear range of amplification (determined independently for each primer set) and products were resolved on 1% agarose gels and stained with Gold View I (Solarbio, Beijing, China). PCR reaction condition 30 cycles of 94 °C for 30 s, 52–55 °C for 30 s, 72 °C for 1 min, followed by 72 °C for 5 min. qRT-PCR was performed using a SYBR^®^ Premix Ex Taq™ II (TaKaRa, Dalian, Liaoning, China) and the ΔΔ*C*_t_ analysis method as Δ*C*_t_ values = *C*_t_ (gene of interest) − *C*_t_ (house-keeping gene), using the constitutively expressed glyceraldehyde 3-phosphate dehydrogenase (GADPH) mRNA for house-keeping gene. All of the qRT-PCR primer sets were designed so the products would span multiple exons, and amplification of a single product of the correct size was confirmed by melting curve analysis. For evaluation of splicing, separate reactions for inclusion and exclusion isoforms were performed for each sample. Primer sequences and cycling conditions were as follows: SIRT1-FL: 5′-ACT GGG GAG AAA AAT GAA AGA A-3′ (forward) and 5′-CTG CCA CAA GAA CTA GAG GAT A-3′ (reverse), Annealing temperature was 55 °C; SIRT1-Δ8: 5′-CAC TAA TTC CAA GTA ATC AGT A-3′ (forward) and 5′-CAC AAG AAC TAG AGG ATA AGA C-3′ (reverse), annealing temperature was 53 °C; HuR(ELAVL1): 5′-AAG CCT GTT CAG CAG CAT TG-3′ (forward) and 5′-CTT CGC GGT CAC GTA GTT CA-3′ (reverse), annealing temperature was 53 °C; TIA1: 5′-TCC CGC TCC AAA GAG TAC ATA TGA G-3′ (forward) and 5′-AAA CAA TTG CAT GTG CTG CAC TTT C-3′ (reverse), annealing temperature was 52 °C; TIAL1: 5′-CAA CTG GAA AAT CCA AAG GCT ATG G-3′ (forward) and 5′-GAC GCA ATT CCT CCA CAG TAC ACA G-3′ (reverse), annealing temperature was 54 °C; GAPDH: 5′-CGC TGA GTA CGT CGT GGA GTC-3′ (forward) and 5′-GCT GAT GAT CTT GAG GCT GTT GTC-3′ (reverse), annealing temperature was 53 °C. Reverse conditions were 95 °C for 30 s, 40 cycles of 95 °C for 5 s, 52–55 °C for 30 s. Results are representative of at least three independent experiments.

### 3.6. Ultraviolet-Induced Stress

The cell culture medium was aspirated and cells were exposed to ultraviolet radiation at 10 j/m^2^ (100 μw/cm^2^ for 10 s). After exposure, 2000 μL DMEM supplemented with 10% fetal bovine serum (FBS) and 1% Pen/Strep was added to the dishes, and they were incubated at 37 °C under 5% CO_2_ for 12 h.

### 3.7. Statistical Analysis

Statistical analyses were performed using SPSS 13.0 (Leians, Beijing, China). Data are expressed as means ± standard error of the mean. All comparisons of means were performed using two-tailed Student’s *t*-test. A *p*-value of 0.05 was set as the threshold for statistical significance. All experiments described in this study were repeated independently at least three times.

## 4. Conclusions

To summarize our study, we found that HuR and TIA1/TIAL1 were involved in regulation of the alternative splicing of SIRT1 pre-mRNA and affect the levels of SIRT1-ΔExon8 under normal and injury circumstances, in which HuR increased SIRT1-ΔExon8 mRNA by promotion of SIRT1 exon 8 exclusion, whereas TIA1/TIAL1 oppositely affected SIRT1-ΔExon8 mRNA by inhibition of this splicing process. Given the diverse properties and functional responses of SIRT1 variants, these regulatory effects of HuR and TIA1/TIAL1 on SIRT1 pre-mRNA alternative splicing might further affect cellular functions especially for the cellular adaptions in environmental stress. In ongoing studies we will emphasize exploration of the mechanisms underlying HuR and TIA1/TIAL1 regulation of SIRT1 pre-mRNA alternative splicing and observe these regulatory effects on cellular functions.
